# Blood Urea Nitrogen and In-Hospital Mortality in Critically Ill Patients with Cardiogenic Shock: Analysis of the MIMIC-III Database

**DOI:** 10.1155/2021/5948636

**Published:** 2021-02-01

**Authors:** En-qian Liu, Chun-lai Zeng

**Affiliations:** Department of Cardiology, Lishui Hospital, Zhejiang University School of Medicine, Lishui, 323000 Zhejiang, China

## Abstract

The association between blood urea nitrogen (BUN) and prognosis has been the focus of recent research. Therefore, the objective of this study was to investigate the association between BUN and hospital mortality in critically ill patients with cardiogenic shock (CS). This was a retrospective cohort study, in which data were obtained from the Medical Information Mart for Intensive Care III V1.4 database. Data from 697 patients with CS were analyzed. Logistic regression and subgroup analyses were used to assess the association between BUN and hospital mortality in patients with CS. The average age of the 697 participants was 71.14 years, and approximately 42.18% were men. In the multivariate logistic regression model, after adjusting for age, sex, diabetes, cardiac arrhythmias, urine output, simplified acute physiology score II, sequential organ failure assessment, creatinine, anion gap, and heart rate, high BUN demonstrated strong associations with increased in-hospital mortality (per standard deviation increase: odds ratio [OR] 1.47, 95% confidence interval [CI] 1.13–1.92). A similar result was observed in BUN tertile groups (BUN 23–37 mg/dL versus 6–22 mg/dL: OR [95% CI], 1.42 [0.86–2.34]; BUN 38–165 mg/dL versus 6–22 mg/dL: OR [95% CI], 1.99 [1.10–3.62]; *P* trend 0.0272). Subgroup analysis did not reveal any significant interactions among various subgroups, and higher BUN was associated with adverse clinical outcomes in patients with CS.

## 1. Introduction

Cardiogenic shock (CS) mainly manifests as hypoperfusion of end organs and hypoxia caused by decreased cardiac output [1, 2]. Acute myocardial infarction (AMI) complicated by left ventricular dysfunction is the most common cause of CS, which is the leading cause of hospital mortality in patients with AMI [3]. In the prospective SHould we emergently revascularize Occluded Coronaries for cardiogenic shocK (SHOCK) Trial Registry, in-hospital mortality in patients with cardiogenic shock (CS) complicating AMI was 60%, and patients with ventricular septal rupture had significantly higher mortality (87.3%) [4]. Patients with CS were at a higher risk of death during the first 4 weeks after admission in the Etude Française de l'Insuffisance Cardiaque Aigue (EFICA) study [5]. However, continuous advances in reperfusion therapy and hospital mortality are still high (27%–51%) [1]. Thus, a simple and convenient method is necessary to stratify patients with CS at high risk of death.

Blood urea nitrogen (BUN) is an indicator of not only renal function but also neurohormonal activation. Several studies have suggested that BUN levels are correlated with short-term, intermediate-term, and long-term prognosis in patients with cardiovascular diseases, including heart failure [6–9], myocardial infarction [10–12], and acute pulmonary embolism [13]. In an observational study, Öz et al. [14] detected that BUN in a high tertile group had a significantly higher incidence of CS. In addition, among 51 patients with ST segment elevation myocardial infarction (STEMI) with profound CS who underwent extracorporeal membrane oxygenation (ECMO) support, Lee et al. [15] noted from multivariate analysis that a higher serum BUN level was associated with increased 30-day mortality. Similarly, Hayıroğlu et al. [16, 17] found an association of BUN with in-hospital mortality and long-term outcome. To the best of our knowledge, few previous studies have evaluated the association between BUN and in-hospital mortality in critically ill patients with CS.

Therefore, this study is aimed at investigating whether BUN was independently related to in-hospital mortality in critically ill patients with CS.

## 2. Materials and Methods

### 2.1. Database Introduction

The Medical Information Mart for Intensive Care III (MIMIC-III) database is an openly available critical care database, which is maintained by the Laboratory for Computational Physiology at the Massachusetts Institute of Technology. Beth Israel Deaconess Medical Center (Boston, Massachusetts, USA) admitted more than 50,000 intensive care unit (ICU) patients from 2001 to 2012 [18]. En-qian Liu completed the online course of the National Institutes of Health and passed the exam for the protection of human research participants (No. 35919439). The institutional review boards of the Massachusetts Institute of Technology (Cambridge, MA) and the Beth Israel Deaconess Medical Center (Boston, MA) approved our use of the database.

### 2.2. Population Selection Criteria

The database included a total of 58,976 ICU admissions. We only analyzed the first ICU stay for patients who were admitted to the ICU more than once. Patients with CS, classified as such using the International Classification of Diseases- (ICD-) 9 code (code = 785.51), were included in our study. We excluded patients who were younger than 16 years of age or who stayed less than 24 h in the ICU as well as those who had solid tumor, metastatic cancer, renal replacement therapy (RRT), liver disease, or no BUN recording.

### 2.3. Data Extraction

Demographic data, clinical data, laboratory data, and scoring systems were extracted using Structured Query Language (SQL) with PostgreSQL (version 9.6). Demographic data included age, sex, and ethnicity. Clinical and laboratory data included heart rate, systolic blood pressure (SBP), diastolic blood pressure (DBP), mean blood pressure (MBP), respiratory rate (RR), temperature, percutaneous oxygen saturation (SpO_2_), urine output, hospital death, renal replacement therapy, vasopressor use, multiple comorbidities, serum sodium, serum potassium, serum creatinine, hemoglobin, serum glucose, anion gap, serum chloride, hematocrit, serum bicarbonate, BUN, platelet count, white blood cell (WBC) count, activated partial thromboplastin time (APTT), prothrombin time (PT), international normalized ratio (INR), sequential organ failure assessment (SOFA), and the simplified acute physiology score II (SAPS II). The laboratory test data were extracted between 6 h before patient ICU admission and the first 24 h afterward. If laboratory examination and vital signs had multiple records, the one correlating with the greatest severity of disease was obtained. Acute kidney injury (AKI) was induced in the first 48 h after patient ICU admission and urine output in the first 24 h. The clinical endpoint was hospital mortality.

### 2.4. Statistical Analysis

All continuous variables were expressed as the mean ± standard deviation (SD) or median (interquartile range [IQR]), and categorical variables were expressed as percentages. Comparisons between the BUN tertile groups were made using the *χ*^2^ test (categorical variables), Fisher's exact test (categorical variables), one-way ANOVA test (normal distribution), or Kruskal-Wallis *H* test (skewed distribution). Patients were divided into tertiles according to BUN values: tertile 1 (6-22 mg/dL), tertile 2 (23-37 mg/dL), and tertile 3 (38-165 mg/dL). A variance inflation factor (VIF) greater than 5 indicated multicollinearity between variables (Supplementary Table S1). Logistic regression was used to define the relationship between BUN levels and in-hospital mortality after correcting for confounding factors. We constructed three models: model 1, where no covariates were adjusted; model 2, adjusted only for age and sex; and model 3, model 2 + other covariates. In order to assess confusion, we entered the covariates into the binary logistic regression model in the basic model, or eliminated the covariates in the complete model one by one, and compared the regression coefficients. Those covariates that changed the initial regression coefficient by more than 10% are also included (Supplementary Table S2). For the sensitivity analysis, we converted BUN into a categorical variable and used a median value in each BUN tertile as a continuous variable to perform the linear trend tests and calculate the *P* trend. Furthermore, interaction and stratified analysis were performed according to age (<75 and ≥75 years), sex, comorbidity (congestive heart failure, cardiac arrhythmias, valvular disease, pulmonary circulation disorder, chronic pulmonary disease, diabetes, hypertension, renal failure, and AKI), and disease severity score (SAPS II and SOFA). Data analysis was performed using the statistical software packages R4.0.2 (http://www.R-project.org, The R Foundation). *P* values greater than 0.05 were considered statistically significant.

## 3. Results

### 3.1. Baseline Characteristics of Selected Participants

**Figure 1:**
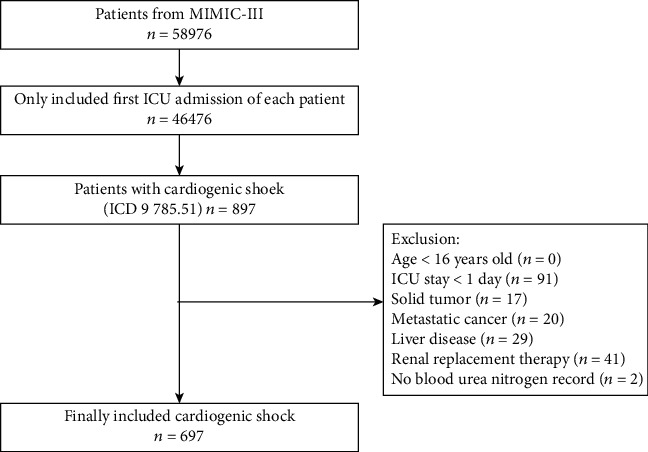
Data filtering flowchart of the study.

In this study, the inclusion criteria were met by 697 participants (see Figure 1 for a flow chart).  Table 1 shows the baseline characteristics of the selected participants grouped by BUN tertile. In general, the average age of the participants was 71.14 ± 13.82 years, and approximately 42.18% of them were men. No statistically significant differences were detected in sex, weight, respiratory rate, temperature, SpO_2_, heart rate, platelet count, APTT, WBC count, hemoglobin, glucose, hematocrit, valvular disease, pulmonary circulation disorder, chronic pulmonary disease, hypertension, ventilation, and vasopressor between different groups (all *P* values > 0.05). Participants with high BUN levels had higher age, platelet, PT, INR, potassium, creatinine, anion gap, SAPS II, and SOFA values. The opposite was observed for MBP, urine output, sodium, chloride, and bicarbonate levels. Patients with congestive heart failure, cardiac arrhythmias, diabetes, renal failure, and AKI were more likely to be in the high BUN group.

### 3.2. Results of Unadjusted and Adjusted Logistic Regression Models

We constructed three different models to analyze the independent effects of BUN on hospital mortality in critically ill patients with CS grouped by BUN tertile.  Table 2 lists the effect sizes (odds ratio [OR] and 95% confidence interval [CI]). The first tertile of BUN was treated as the reference group. In the crude model, BUN per SD increase was associated with hospital mortality (OR 1.70, 95% CI 1.44–2.01). After adjustment for age and sex, the results did not demonstrate any obvious change (OR 1.64, 95% CI 1.39–1.95). Similarly, the correlations were significant after adjusting for age, sex, diabetes, cardiac arrhythmias, urine output, SAPS II, SOFA, creatinine, anion gap, and heart rate (OR 1.47, 95% CI 1.13–1.92). For sensitivity analysis, BUN was converted into tertiles. After adjusting for age and sex, compared with those of the reference group (BUN 6–22 mg/dL), the adjusted ORs (95% CIs) for BUN levels 23–37 mg/dL and 38–165 mg/dL were 2.09 (1.32–3.30) and 3.56 (2.25–5.64), respectively. After adjusting for age, sex, diabetes, cardiac arrhythmias, urine output, SAPS II, SOFA, creatinine, anion gap, and heart rate, high BUN maintained strong associations with increased hospital mortality (BUN 23–37 mg/dL versus 6–22 mg/dL: OR [95% CI], 1.42 [0.86–2.34]; BUN 38–165 mg/dL versus 6–22 mg/dL: OR [95% CI], 1.99 [1.10–3.62]; *P* trend 0.0272).

### 3.3. The Results of Subgroup Analyses

**Figure 2:**
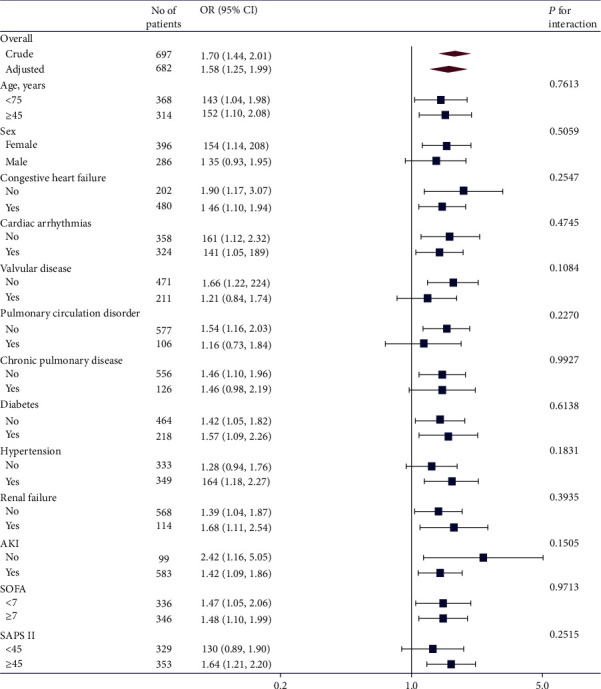
Effect size of BUN on hospital mortality in prespecified and exploratory subgroups. Adjusted for age, sex, diabetes, cardiac arrhythmias, urine output, SAPS II, SOFA, creatinine, anion gap, and heart rate, except the subgroup variable.

As shown in Figure 2, we performed an interaction and stratified analysis to assess the association between BUN and hospital mortality. The interaction was not significant according to age (<75 and ≥75 years), sex, comorbidity (congestive heart failure, cardiac arrhythmias, valvular disease, pulmonary circulation disorder, chronic pulmonary disease, diabetes, hypertension, renal failure, and AKI), and disease severity score (SAPS II and SOFA). The effect sizes of BUN on mortality were significant in different subgroups, and there were no significant interactions.

## 4. Discussion

This study was performed to evaluate the association between BUN and hospital mortality in critically ill patients with cardiogenic shock. Our study indicated that BUN, as a categorical or continuous variable, significantly correlated with hospital mortality in multivariate logistic regression analysis. Consistent results were observed in the stratified analysis.

Urea is freely filtered at the glomerulus and reabsorbed by the renal tubules in the kidneys. Urea reabsorption occurs through two mechanisms: the concentration-dependent reabsorption in the proximal tubules and arginine vasopressin- (AVP-) dependent reabsorption in the collecting duct [19, 20]. Activation of the sympathetic and renin-angiotensin-aldosterone systems lowers urine flow rates to increase urea's concentration-dependent reabsorption [20]. In addition, arginine-vasopressin systems act on urinary aquaphorin-2 water channels to enhance urea's reabsorption in the collecting tubules [20]. Therefore, BUN not only reflects renal function but is also related to neurohormonal activation. The typical characteristics of CS are persistent hypotension and end-organ hypoperfusion [1]. The baroreceptors in the neck sense hypotension and hypovolemia and subsequently promote AVP secretion to maintain the stability of blood circulation [21]. Thus, increased AVP may result in increased BUN reabsorption in the collecting duct. When renal hypoperfusion occurs in patients with CS, BUN increases with decreasing glomerular filtration rate. For the above reasons, BUN may be associated with CS; however, the association between BUN and in-hospital mortality in critically ill patients with CS is unclear. To the best of our knowledge, in a retrospective cohort study of 252 consecutively confirmed acute pulmonary embolism patients treated with tissue plasminogen activator (t-PA), patients in the high BUN group (≥34.5 mg/dL) had a higher incidence of CS, and elevated admission BUN levels were independently related with a high risk of in-hospital mortality [13]. The Acute Decompensated Heart Failure Syndromes (ATTEND) registry cohort of 4,449 patients with acute decompensated heart failure described the association between eGFR at discharge and the risk of all-cause mortality that was modified by BUN at discharge [7]. Additionally, elevated BUN levels on admission were associated with worse long-term cardiovascular mortality in 1,332 consecutive acute myocardial infarction patients and may help to identify high-risk patients [10]. On the other hand, among 26,288 critically ill patients in a cohort study, Beier et al. [22] proposed that BUN was associated with short-term and long-term mortality, independent of serum creatinine (0.8–1.3 mg/dL). Recently, in a cohort investigating patients with STEMI complicated by CS, Hayıroğlu et al. [16, 17] reported an association between a higher BUN and increased risk of in-hospital mortality (OR 1.06, 95% CI 1.03–1.09) and long-term mortality (hazard ratio [HR] 1.019, 95% CI 1.005–1.034) in the fully adjusted models. Furthermore, our results showed that in critically ill patients with CS, increased BUN was related to in-hospital mortality (BUN per SD increase: OR 1.47, 95% CI 1.13–1.92). In addition, the association existed stably in different subgroups according to age (<75 and ≥75 years), sex, comorbidities (congestive heart failure, cardiac arrhythmias, valvular disease, pulmonary circulation disorder, chronic pulmonary disease, diabetes, hypertension, renal failure, and AKI), and disease severity score (SAPS II and SOFA). BUN levels may help to identify high-risk patients. Evidently, large prospective and multicentric studies are paramount to substantiate the association between BUN and adverse outcomes in patients with CS.

Our study had several strengths, including the following: (1) our study observed the association between BUN and hospital mortality in critically ill patients with CS and (2) the findings of this study may be beneficial to the clinician in identifying high-risk patients with CS.

Some limitations to this study included the following: (1) our research patients were critically ill patients with CS who had no cancer, liver disease, or RRT; therefore, this conclusion is not suitable for extrapolation to other groups; (2) BUN could have been affected by many cofounders, such as dietary patterns and kinds of drugs; however, this database analysis was retrospective, and these situations could not be distinguished; and (3) because this database analysis was a single-center research and contained some inaccurate information, a multicenter prospective research is necessary to verify our conclusions.

## 5. Conclusions

In critically ill patients with CS, higher BUN, as a continuous or categorical variable, was associated with adverse clinical outcomes after adjusting for age, sex, diabetes, cardiac arrhythmias, urine output, SAPS II, SOFA, creatinine, anion gap, and heart rate.

## Figures and Tables

**Table 1:** Characteristics of the study patients according to blood urea nitrogen level.

BUN (mg/dL) tertile	All patients	T1 (6-22)	T2 (23-37)	T3 (38-165)	*P* value
Number, *n*	697	214	247	236	
Age (years)	71.14 ± 13.82	64.53 ± 14.82	72.75 ± 12.10	75.46 ± 12.31	<0.001
Sex, *n* (%)					0.191
Female	403 (57.82)	126 (58.88)	132 (53.44)	145 (61.44)	
Male	294 (42.18)	88 (41.12)	115 (46.56)	91 (38.56)	
Ethnicity, *n* (%)					0.002
White	453 (64.99)	125 (58.41)	172 (69.64)	156 (66.10)	
Black	36 (5.16)	16 (7.48)	10 (4.05)	10 (4.24)	
Unknown	165 (23.67)	51 (23.83)	61 (24.70)	53 (22.46)	
Other	43 (6.17)	22 (10.28)	4 (1.62)	17 (7.20)	
ICU type, *n* (%)					<0.001
CCU	432 (61.98)	129 (60.28)	156 (63.16)	147 (62.29)	
CSRU	119 (17.07)	56 (26.17)	44 (17.81)	19 (8.05)	
MICU	115 (16.50)	17 (7.94)	37 (14.98)	61 (25.85)	
SICU/TSICU	31 (4.45)	12 (5.61)	10 (4.05)	9 (3.81)	
Weight (kg)	80.33 ± 20.86	80.22 ± 20.69	79.15 ± 20.60	81.69 ± 21.31	0.416
MBP (mmHg)	52.93 ± 13.64	56.38 ± 13.72	52.12 ± 13.78	50.65 ± 12.82	<0.001
Respiratory rate (beats/minute)	28.33 ± 7.36	27.55 ± 7.06	28.34 ± 7.87	29.03 ± 7.02	0.106
Temperature (°C)	35.85 ± 1.09	35.95 ± 1.21	35.78 ± 1.09	35.84 ± 0.97	0.278
SpO_2_ (%)	88.63 ± 12.24	89.72 ± 10.66	88.91 ± 11.77	87.35 ± 13.89	0.113
Heart rate (beats/minute)	110.59 ± 22.48	109.79 ± 19.95	111.40 ± 22.98	110.47 ± 24.14	0.745
Urine output (mL/24 h)	1969.13 ± 1378.83	2557.31 ± 1535.30	1920.77 ± 1202.52	1480.20 ± 1190.31	<0.001
Platelet (K/*μ*L)	199.26 ± 95.14	202.78 ± 107.54	191.13 ± 84.28	204.53 ± 93.66	0.245
PT (second)	18.90 ± 12.73	16.18 ± 4.63	17.94 ± 9.08	22.35 ± 18.79	<0.001
INR	2.02 ± 2.03	1.60 ± 0.77	1.87 ± 1.46	2.54 ± 2.99	<0.001
APTT (second)	76.01 ± 44.20	75.04 ± 44.38	79.23 ± 43.92	73.47 ± 44.31	0.340
WBC (10^9^/L)	16.34 ± 6.84	16.06 ± 7.05	16.47 ± 6.09	16.46 ± 7.38	0.771
Hemoglobin (g/dL)	10.26 ± 2.32	10.50 ± 2.52	10.16 ± 2.47	10.16 ± 1.92	0.206
Glucose (mg/dL)	125.18 ± 47.06	122.80 ± 46.62	125.81 ± 45.81	126.69 ± 48.82	0.659
Sodium (mEq/L)	139.84 ± 4.49	140.26 ± 4.33	140.05 ± 4.44	139.24 ± 4.64	0.036
Potassium (mEq/L)	3.70 ± 0.61	3.48 ± 0.53	3.59 ± 0.50	4.03 ± 0.64	<0.001
Hematocrit (%)	30.24 ± 6.64	30.52 ± 7.26	29.86 ± 6.89	30.39 ± 5.73	0.511
Chloride (mEq/L)	106.87 ± 6.25	108.04 ± 6.17	107.19 ± 6.37	105.48 ± 5.94	<0.001
Creatinine (mg/dL)	1.74 ± 1.21	1.02 ± 0.32	1.46 ± 0.52	2.69 ± 1.58	<0.001
Bicarbonate (mEq/L)	19.84 ± 4.71	20.51 ± 4.46	20.08 ± 4.47	18.99 ± 5.05	0.002
Anion gap (mEq/L)	18.29 ± 4.59	16.60 ± 3.55	17.65 ± 3.93	20.46 ± 5.18	<0.001
SAPS II	45.77 ± 14.95	37.20 ± 13.06	46.63 ± 13.55	52.64 ± 14.15	<0.001
SOFA	6.56 ± 3.52	5.37 ± 3.51	6.70 ± 3.42	7.50 ± 3.33	<0.001
Congestive heart failure, *n* (%)	490 (70.30)	133 (62.15)	171 (69.23)	186 (78.81)	<0.001
Cardiac arrhythmias, *n* (%)	330 (47.35)	80 (37.38)	111 (44.94)	139 (58.90)	<0.001
Valvular disease, *n* (%)	220 (31.56)	60 (28.04)	83 (33.60)	77 (32.63)	0.400
Pulmonary circulation disorder, *n* (%)	108 (15.49)	27 (12.62)	37 (14.98)	44 (18.64)	0.203
Chronic pulmonary disease, *n* (%)	128 (18.36)	30 (14.02)	45 (18.22)	53 (22.46)	0.069
Diabetes, *n* (%)	225 (32.28)	40 (18.69)	80 (32.39)	105 (44.49)	<0.001
Hypertension, *n* (%)	360 (51.65)	102 (47.66)	132 (53.44)	126 (53.39)	0.374
Renal failure, *n* (%)	118 (16.93)	6 (2.80)	31 (12.55)	81 (34.32)	<0.001
AKI, *n* (%)	596 (85.51)	167 (78.04)	214 (86.64)	215 (91.10)	<0.001
Ventilation, *n* (%)	453 (64.99)	134 (62.62)	173 (70.04)	146 (61.86)	0.116
Vasopressor, *n* (%)	516 (74.03)	150 (70.09)	187 (75.71)	179 (75.85)	0.288

Notes: data are presented as the mean ± SD, median (Q1–Q3), or *N* (%). MBP: mean blood pressure; BUN: blood urea nitrogen; WBC: white blood cell; PT: prothrombin time; APTT: activated partial thromboplastin time; INR: international normalized ratio; SOFA: sequential organ failure assessment; SAPS II: simplified acute physiology score II; AKI: acute kidney injury.

**Table 2:** Relationship between BUN and in-hospital mortality in different models.

	Model 1	Model 2	Model 3
	OR (95% CI), *P* value	OR (95% CI), *P* value	OR (95% CI), *P* value
BUN per SD increase	1.70 (1.44, 2.01), <0.0001	1.64 (1.39, 1.95), <0.0001	1.47 (1.13, 1.92), 0.0041
BUN tertile			
T1	Ref	Ref	Ref
T2	2.41 (1.54, 3.77), 0.0001	2.09 (1.32, 3.30), 0.0016	1.42 (0.86, 2.34), 0.1765
T3	4.17 (2.69, 6.48), <0.0001	3.56 (2.25, 5.64), <0.0001	1.99 (1.10, 3.62), 0.0232
*P* for trend	<0.0001	<0.0001	0.0272

OR: odds ratio; CI: confidence interval; Ref: reference. Model 1 was adjusted for none, Model 2 for age and sex, and Model 3 for age, sex, diabetes, cardiac arrhythmias, urine output, SAPS II, SOFA, creatinine, anion gap, and heart rate.

## Data Availability

Answer: Yes. Comment: The corresponding author can be contacted to obtain the data used in this study (zengchunlai@aliyun.com). However, reanalysis of the complete data requires approval from the MIMIC III Institute.

## Supplementary Materials

Supplementary Materials The supplementary material file contains the following: (1) Table S1: results of the variance inflation factor (VIF). (2) Table S2: associations of covariates with in-hospital mortality.
