# Supplementary material for: Blood Urea Nitrogen and In-Hospital Mortality in Critically Ill Patients with Cardiogenic Shock: Analysis of the MIMIC-III Database
Source: Biomed Res Int. 2021 Feb 1;2021:5948636. doi: 10.1155/2021/5948636 (PMC7870297; doi:10.1155/2021/5948636)
Supplement: Supplementary Materials — The supplementary material file contains the following: (1) Table S1: results of the variance inflation factor (VIF). (2) Table S2: associations of covariates with in-hospital mortality. [file 5948636.f1.docx]

## Table S1: Results of the variance inflation factor (VIF)

|  | Step 1 | Step 2 | Step 3 |
| --- | --- | --- | --- |
| BUN | 3 | 2.9 | 2.9 |
| Age | 1.8 | 1.8 | 1.8 |
| Sex | 1.4 | 1.4 | 1.3 |
| Ethnicity | 1.1 | 1.1 | 1.1 |
| ICU type | 1.2 | 1.2 | 1.2 |
| Congestive heart failure | 1.2 | 1.2 | 1.2 |
| Cardiac arrhythmias | 1.2 | 1.2 | 1.2 |
| Valvular disease | 1.2 | 1.2 | 1.2 |
| Pulmonary circulation disorder | 1.2 | 1.1 | 1.1 |
| Chronic pulmonary disease | 1.1 | 1.1 | 1.1 |
| Diabetes | 1.2 | 1.2 | 1.2 |
| Hypertension | 1.3 | 1.2 | 1.2 |
| Renal failure | 1.6 | 1.5 | 1.5 |
| SAPSII | 4.2 | 4.2 | 4.2 |
| AKI | 1.2 | 1.2 | 1.2 |
| Weight, kg | 1.6 | 1.6 | 1.6 |
| Urine output, mL | 1.6 | 1.6 | 1.6 |
| Platelet, K/uL | 1.5 | 1.5 | 1.5 |
| PT, second | 4 | 4 | 3.9 |
| WBC, 10^9^/L | 1.4 | 1.4 | 1.4 |
| Hemoglobin, g/dL* | 17.7 | NA | NA |
| Glucose, mg/dL | 1.2 | 1.2 | 1.2 |
| Sodium, mEq/L | 4.1 | 4.1 | 1.3 |
| INR | 3.8 | 3.8 | 3.7 |
| Potassium, mEq/L | 1.7 | 1.6 | 1.6 |
| APPT, second | 1.3 | 1.3 | 1.3 |
| Hematocrit, % | 17.5 | 1.5 | 1.5 |
| Chloride, mEq/L* | 6 | 6 | NA |
| Creatinine, mg/dL | 3 | 3 | 3 |
| Bicarbonate, mEq/L | 4.4 | 4.4 | 2.2 |
| Anion gap, mEq/L | 3.5 | 3.4 | 2.2 |
| Ventilation | 1.9 | 1.9 | 1.8 |
| Heart rate, beats/minute | 1.3 | 1.3 | 1.3 |
| MBP, mmHg | 1.4 | 1.4 | 1.4 |
| Respiratory rate, beats/minute | 1.2 | 1.2 | 1.2 |
| Temperature, °C | 1.4 | 1.4 | 1.4 |
| SPO2, % | 1.2 | 1.2 | 1.2 |
| Vasopressor | 1.9 | 1.9 | 1.9 |
| SOFA | 4 | 4 | 4 |

## *Variables eliminated by collinearity screening

## Table S2: Associations of covariates with in-hospital mortality.

| Variables | Basic model |  | Complete model | Select |
| --- | --- | --- | --- | --- |
|  | BUN(mg/dL) |  | BUN(mg/dL) |  |
|  |  |  |  |  |
| Initial regression coefficient | 0.0208 |  | 0.0157 |  |
|  |  |  |  |  |
| Ethnicity | 0.0215 |  | 0.0169 |  |
|  |  |  |  |  |
| ICU type | 0.0192 |  | 0.0161 |  |
|  |  |  |  |  |
| Congestive heart failure | 0.0221 |  | 0.0145 |  |
|  |  |  |  |  |
| Cardiac arrhythmias | 0.0216 |  | 0.0141 * | Yes |
|  |  |  |  |  |
| Valvular disease | 0.0209 |  | 0.0157 |  |
|  |  |  |  |  |
| Pulmonary circulation disorder | 0.0208 |  | 0.0160 |  |
|  |  |  |  |  |
| Chronic pulmonary disease | 0.0206 |  | 0.0156 |  |
|  |  |  |  |  |
| Diabetes | 0.0239 * |  | 0.0139 * | Yes |
|  |  |  |  |  |
| Hypertension | 0.0218 |  | 0.0170 |  |
|  |  |  |  |  |
| Renal failure | 0.0225 |  | 0.0165 |  |
|  |  |  |  |  |
| SAPSII | 0.0129 * |  | 0.0173 * | Yes |
|  |  |  |  |  |
| AKI | 0.0200 |  | 0.0154 |  |
|  |  |  |  |  |
| Weight, kg | 0.0208 |  | 0.0158 |  |
|  |  |  |  |  |
| Urine output, mL | 0.0163 * |  | 0.0149 | Yes |
|  |  |  |  |  |
| Platelet, K/uL | 0.0208 |  | 0.0156 |  |
|  |  |  |  |  |
| PT, second | 0.0200 |  | 0.0148 |  |
|  |  |  |  |  |
| WBC, 10^9^/L | 0.0205 |  | 0.0153 |  |
|  |  |  |  |  |
| Glucose, mg/dl | 0.0207 |  | 0.0153 |  |
|  |  |  |  |  |
| Sodium, mEq/L | 0.0208 |  | 0.0157 |  |
|  |  |  |  |  |
| INR | 0.0198 |  | 0.0151 |  |
|  |  |  |  |  |
| Potassium, mEq/L | 0.0194 |  | 0.0168 |  |
|  |  |  |  |  |
| APPT, second | 0.0210 |  | 0.0157 |  |
|  |  |  |  |  |
| Hematocrit, % | 0.0208 |  | 0.0155 |  |
|  |  |  |  |  |
| Creatinine, mg/dL | 0.0152 * |  | 0.0105 * | Yes |
|  |  |  |  |  |
| Bicarbonate, mEq/L | 0.0188 |  | 0.0159 |  |
|  |  |  |  |  |
| Anion gap, mEq/L | 0.0138 * |  | 0.0171 | Yes |
|  |  |  |  |  |
| Ventilation | 0.0214 |  | 0.0158 |  |
|  |  |  |  |  |
| Heart rate, beats/minute | 0.0219 |  | 0.0133 * | Yes |
|  |  |  |  |  |
| MBP, mmHg | 0.0200 |  | 0.0155 |  |
|  |  |  |  |  |
| Respiratory rate, beats/minute | 0.0203 |  | 0.0158 |  |
|  |  |  |  |  |
| Temperature, °C | 0.0207 |  | 0.0157 |  |
|  |  |  |  |  |
| S_P_O_2_, % | 0.0204 |  | 0.0155 |  |
|  |  |  |  |  |
| Vasopressor | 0.0205 |  | 0.0148 |  |
|  |  |  |  |  |
| SOFA | 0.0164 * |  | 0.0143 | Yes |
|  |  |  |  |  |

## * Indicates a change of more than 10% compared to the initial regression coefficient
